# Sequence polymorphisms of *rfbT* among the *Vibrio cholerae* O1 strains in the Ogawa and Inaba serotype shifts

**DOI:** 10.1186/1471-2180-13-173

**Published:** 2013-07-26

**Authors:** Weili Liang, Luxi Wang, Pu Liang, Xiao Zheng, Haijian Zhou, Jingyun Zhang, Lijuan Zhang, Biao Kan

**Affiliations:** 1 State Key Laboratory for Infectious Disease Prevention and Control, National Institute for Communicable Disease Control and Prevention, Chinese Center for Disease Control and Prevention, Beijing, 102206, People’s Republic of China; 2 Collaborative Innovation Center for Diagnosis and Treatment of Infectious Diseases, Hangzhou, People’s Republic of China; 3 Present address: Changping Center for Disease Control and Prevention, Beijing, People’s Republic of China

## Abstract

**Background:**

*Vibrio cholerae* serogroup O1 has two major serotypes, Ogawa and Inaba, which may alternate among cholera epidemics. The *rfbT* gene is responsible for the conversion between the two serotypes. In this study, we surveyed the sequence variance of *rfbT* in the Ogawa and Inaba strains in China over a 48-year (1961-2008) period in which serotype shifts occurred among epidemic years.

**Results:**

Various mutation events including single nucleotide, short fragment insertions/deletions and transposases insertions, were found in the *rfbT* gene of the Inaba strains. Ectopically introducing an intact *rfbT* could overcome the mutations by converting the Inaba serotype to the Ogawa serotype, suggesting the effects of these mutations on the function of RfbT. Characteristic *rfbT* mutations were recognized in the Inaba strains among Inaba serotype dominant epidemic years which were separate from the Ogawa dominant epidemics. Three distinguishable mutation sites in *rfbT* between the classical and the El Tor biotype strains were identified and could serve as biotype-specific biomarkers.

**Conclusions:**

Our results provide a comprehensive picture of the *rfbT* gene mutations among the *V. cholerae* O1 strains in different epidemic periods, which could be further used as the tracing markers in clonality analysis and dissemination surveillance of the epidemic strains.

## Background

Cholera is a severe disease characterized by watery diarrhea that is caused by the gram-negative bacterium *V. cholerae*. The massive diarrhea experienced by patients is mainly due to the colonization of toxigenic *V. cholerae* strains in the small intestine and their production of cholera toxin (CT) [1]. Cholera continues to be a major public health concern in many developing countries [2,3]. Outbreaks of cholera have been increasing globally in the past decade, most recently in Haiti [4].

*V. cholerae* is naturally present in the environment and autochthonous to coastal and estuarine ecosystems. Based on the heat-stable somatic O antigen, the species *V. cholerae* is divided into more than 200 serogroups [5,6]. Only two serogroups, O1 and O139, have thus far been demonstrated to cause epidemic and pandemic cholera. Seven pandemics caused by *V. cholerae* O1 have been reported since 1871. *V. cholerae* O139 emerged in late 1992 on the India subcontinent [7,8]. *V. cholerae* O1 exists as two biotypes, classical and El Tor, which are distinguished by a variety of phenotypic markers, and differ in the severity of their infections and ability to survive outside the human intestine as well [3,9-11]. Two of the first six cholera pandemics are known to have been caused by the classical biotype, while the ongoing seventh pandemic, which began in 1961, is caused by the El Tor biotype.

The vast majority of strains within the O1 serogroup display one of two serotypes, Ogawa or Inaba. A third serotype called Hikojima also exists, but is rare and unstable and not recognized by some authorities [3]. The Ogawa and Inaba serotypes differ by the presence of a 2-*O-*methyl group in the nonreducing terminal carbohydrate in the Ogawa O antigen [12,13]. The O antigen is not a primary gene product, but rather, an assemblage of sugar moieties. The genes responsible for the synthesis of the O1-specific antigens are present in a cluster designated the *rfb* region [14]. Genetic changes in this region are correlated with specific somatic antigens which are serologically different. The serogroup O139 resulted from a 22 kb deletion of the *rfb* region of an O1 El Tor strain, with replacement by a 35 kb *wbf* region encoding the O139 specific O antigen [15].

Serotype conversion within the O1 serogroup has been demonstrated to occur during subculture *in vitro*, passage *in vivo*, epidemics and during phage treatment [16-21]. Genetic alterations in the *rfbT* gene account for the serotype shift which encodes a transferase responsible for the expression of the Ogawa-specific antigen [19,22,23]. Site-specific sequence mutations causing a frameshift in the *rfbT* gene, thus producing truncated RfbT proteins, were previously detected in Inaba strains [19,22,24]. Generally, the serotype shift occurs more frequently in the direction of Ogawa to Inaba [3]. Experimentally providing the intact *rfbT* gene can convert the Inaba serotype to the Ogawa serotype, but this cannot be achieved during the natural infection process. Theoretically, one Ogawa strain may arise from the reversion of an original mutation, but the correction of the specific substitution or deletion is necessarily a rare event [3,22]. Mutations in *rfbT* were used to assess the clonal origin and dissemination of clinical Inaba isolates [24]. The serotype shift pattern of cholera in endemic areas was also historically observed [25,26] and indicated to be associated with high, but incomplete, cross-immunity between the Ogawa and Inaba serotypes [20].

Continuous surveys on the Inaba strains may reveal more mutations of the *rfbT* gene, and even clonality of the epidemic *V. cholerae* strains. In China the seventh cholera pandemic caused by O1 El Tor *V. cholerae* started in July 1961 [27]. Notifiable cases of cholera reported to the national disease surveillance and reporting system showed that there were serotype shifts during the years of El Tor biotype epidemics. In this study, diversity of the *rfbT* sequence and the effect of the *rfbT* mutations on the serotyping were investigated. Characteristic mutations causing serotype shifts in different Inaba predominant epidemics were observed.

## Methods

### Bacteria strains, media and plasmids

This study was conducted on 134 O1 El Tor and 1 O1 classical *V. cholerae* strains isolated from different provinces in China from 1961 to 2008,together with 18 laboratory-collected O1 classical strains and 10 O1 El Tor strains isolated outside of China (Additional file 1: Table S1). All strains were recovered from −80°C laboratory stocks. Slide agglutination tests were used to serotype the strains using anti-Ogawa and anti-Inaba monoclonal antibodies (S&A reagents lab, Bangkok, Thailand). Classical biotype strains were further confirmed using the Classical IV bacteriophage susceptibility assay [28] and the polymyxin B (50U) susceptibility assay with *V. cholerae* 569B and N16961 used as reference strains. The pBR322 plasmid was used as the cloning vector. Suicide plasmid pCVD442 was used to engineer mutations in host strains via allelic exchange. *Escherichia coli* strain Top10 and SM10λ*pir* were used as the recipient strains. All strains were grown in Luria-Bertani (LB) broth or Luria-Bertani (LB) agar plates at 37°C. Ampicillin was used at a final concentration of 100 μg/ml when necessary.

### PCR amplification and construction of complementary plasmid

PCR amplification was carried out using standard protocols with rfbt-up (5′ GCG TCG ACG AAT CGG CAG TCG CAA CA 3′) and rfbt-dn (5′ CCC AAG CTT CAA AGC TAT ACT AAA CTG 3′) primers. A water-boiled template of each strain was used. The 1441 bp PCR products were purified with a QIAGEN PCR purification kit (Qiagen Inc., Hilden, Germany) and applied for commercial sequencing. The nucleotide and deduced protein sequences were analyzed with the MegAlign program included in the DNASTAR software package (DNA Star Inc., Madison, WI). The PCR product of *rfbT* from Ogawa strain O395 was cloned into pBR322 after gel purification and cleavage with *Sal*I and *Hind*III. The resulting plasmid, pBR322-*rfbT*, expressed the *rfbT* gene from its own promoter.

### Construction of mutant

T472C substitution mutant was constructed by allelic exchange using Ogawa strain 7743 as a wild-type precursor which was an ideal natural vaccine candidate strain selected in our laboratory previously [29,30]. The target sequences was amplified with primer pair rfbT-472C-up-*Sal*I/rfbT-472C-dn-*Sac*I (5′ AAC *GTC GAC* GAG GTA GTA ATG AAA CAT CT 3′/5′ C*GA GCT C*AG GAA TTC ACA GCA CAT C 3′, in which the nucleotides in italics indicate the restriction sites) using strain ZJ05023 as the template which contains T472C substitution on the chromosomal *rfbT* gene. The 978 bp amplification product was directionally cloned into pUC19 using *E. coli* TOP10 as the host and confirmed by sequencing of both DNA strands with M13 forward and reverse primers. The corresponding *Sal*I/*Sac*I fragment was subsequently subcloned into suicide plasmid pCVD442. The resulting suicide plasmid was constructed in *E. coli* SM10λ*pir* and mobilized into Ogawa strain 7743 by conjugation. Exconjugants were selected on LB agar containing PolB (100 unit/ml) and Amp (150 μg/ml) and streaked on LB agar containing 15% (w/v) sucrose. Sucrose-resistant colonies were tested for Amp sensitivity and then screened for serotype conversion with slide agglutination tests. The colonies displaying Inaba serotype was confirmed by DNA sequencing using primers rfbT-472C-up-*Sal*I and rfbT-472C-dn-*Sac*I.

### Gene complementation

*rfbT* complementation tests were performed by introducing the *rfbT*-expressing plasmid pBR322-*rfbT* into selected *V. cholerae* Inaba strains by electroporation as described by Chiang and Mekalanos [31]. Overnight cultures from fresh single colonies were subcultured 1:100 in LB and grown to mid-log phase at 37°C on a roller shaker. Cells from 5 ml of a mid-log-phase culture were washed three times in 2.5 ml of ice-cold 2 mM CaCl_2,_ and then resuspended in 100 μl of ice-cold 2 mM CaCl_2_. The electroporated cells were recovered at 30°C for 2 h without shaking and plated on LB agar containing ampicillin (100 μg/ml). Colonies from each electroporation were re-streaked on LB agar containing ampicillin and used to screen for serotype conversion with slide agglutination tests.

### Pulsed-field gel electrophoresis (PFGE)

The PFGE analysis was conducted as described in the literature [32]. Briefly, cell suspensions were adjusted to an optical density of 4.0–4.2 using the Densimat photometer (BioMérieux, Marcy l’Etoile, France). Agarose plugs were prepared, and the organisms in the plugs were digested using 20 U per slice of *Not*I. Electrophoresis was performed using a CHEF-DRIII system (Bio-Rad Laboratories, Hercules, CA). The BioNumerics software package (version 4.0; Applied Maths, Inc.) was used to analyze the PFGE patterns. A dendrogram was produced using the Dice coefficient and the unweighted-pair group method with an arithmetic mean algorithm (UPGMA) with a position tolerance of 1.3%.

### Nucleotide sequences and accession numbers

The *rfbT* genes with sequence variation from the Chinese strains were deposited in the NCBI database under accession numbers JX565645-JX565687, respectively. The *rfbT* sequences of strains N16961 [33], MJ-1236 [34], M66-2 [35], 2010EL-1786 [36], RC9 (accession number ACHX01000006.1), B33 [34], CIRS101 [34], IEC224 [37], LMA3984-4 [38] and NIH35A3 (accession number X59779) were downloaded from the NCBI database.

## Results

### Serotype shifts during the cholera epidemics in China

**Figure 1:**
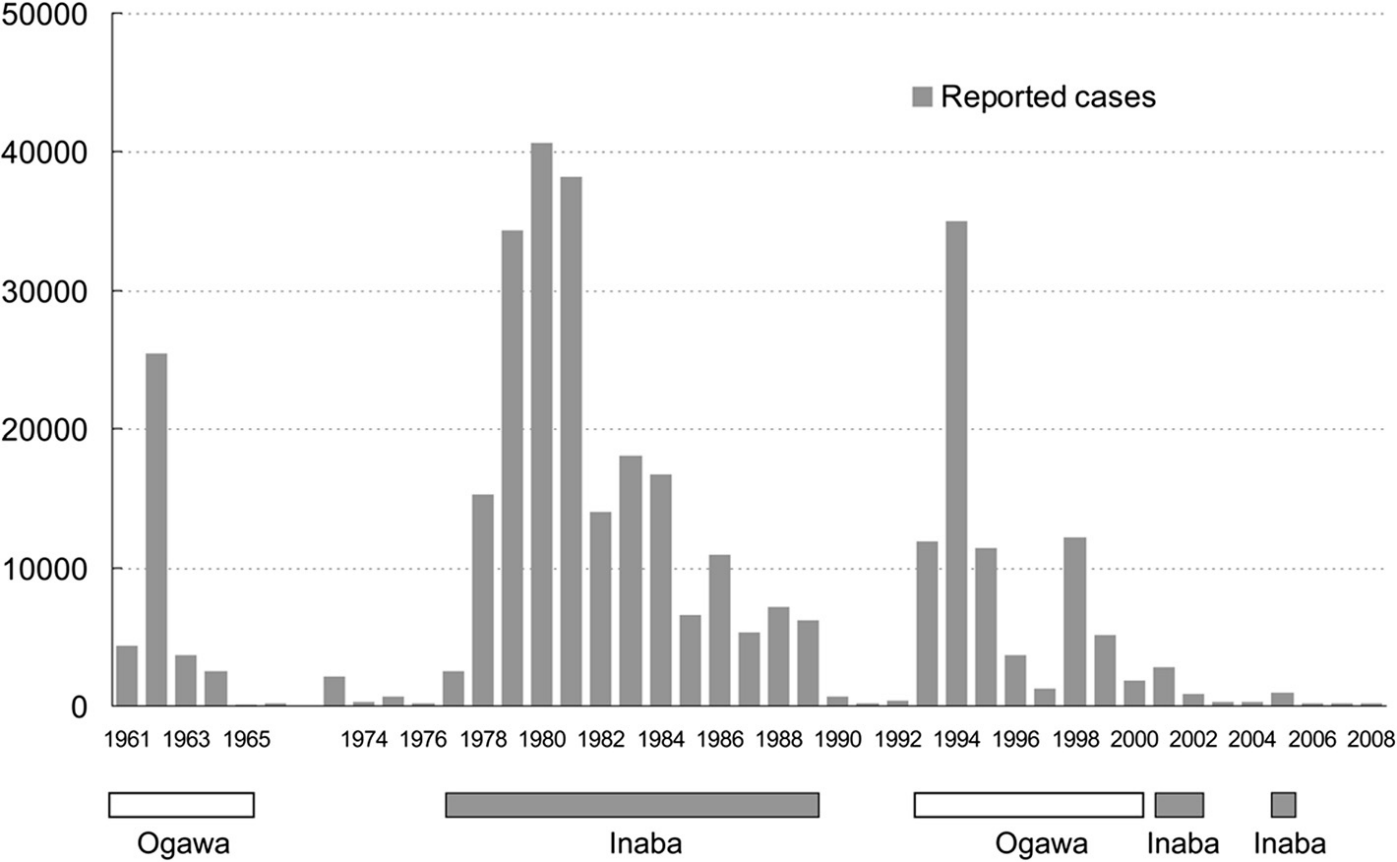
**Reported cases in the cholera surveillance of China and the dominant serotypes of *V. cholerae *O1 strains during the different epidemic years.**

Based on the surveillance data, cholera epidemics in China can be recognized as occurring in three different periods, with peaks of reported cases in 1962, 1980 and 1994, and the intervening periods respectively [39,40]. As shown in Figure 1, the Ogawa serotype dominated during the first epidemic period from 1961 to 1964, while the Inaba dominated the second epidemic period from 1978 to 1989. During the third epidemic period from 1993 to 2000, Ogawa reemerged as the dominant serotype, although a new serogroup, O139, emerged in 1993. Each transition of the dominant serotype was followed by the appearance of a new epidemic peak. After 2000, cholera subsided to a very low level of epidemic, but serotype shifts were still observed. The Inaba serotype significantly increased in 2001 and 2002 after having almost disappeared for ten years. The Inaba serotype upsurged in 2005 and decreased in 2006.

### Sequence variations in Ogawa serotype strains

A previous study with a very limited number of strains showed no significant sequence mutations in the Ogawa serotype [22]. Here we sequenced the *rfbT* genes of 71 Ogawa isolates, including 6 classical strains and 65 El Tor strains (Additional file 1: Table S1). Except strains 6310, 6312 and 63–12 (from Indonesia), 863 (from Mauritania) and C7258 (from Peru), the El Tor strains were isolated from 13 different provinces in China over a 44-year period. In addition, the *rfbT* sequences of four whole genome-sequenced Ogawa strains, M66-2 (from Indonesia in 1937, a pre-seventh pandemic strain) [35], B33 (from Mozambique in 2004) [34], RC9 (from Kenya in 1985, accession number ACHX01000006) and 2010EL-1786 (from Haiti in 2010) [36], were retrieved from the NCBI database. The ORF of *rfbT* (Vch1786_I2540) in 2010EL-1786 was recognized as a fragment of 903bp in its annotation file. After carefully examined the sequence, we revised the sequence by removing the additional 42 bps from the 5′ side (positions 2687324–2687365 in the genomic sequence of NC_016445.1) in our analysis.

The sequence alignments of 75 Ogawa strains revealed high homology in *rfbT* nucleotide sequence except for three sites which were different, but conserved between classical and El Tor biotypes respectively. All 69 El Tor biotype Ogawa strains had identical sequences. Compared with the sequences of El Tor biotype, substitutions of T for G at position 137 (G137T), TACA303-306ACAC (as the result of T-303 deletion and a C insertion after C-307 in the classical Ogawa strains) and C487A were found in all six classical Ogawa strains (Additional file 2: Figure S1), which resulted in amino acid changes of W46L, T102H and Q163K, respectively. Strain 16503 has another mutation G456A compared with all other Ogawa strains. Since all the strains are Ogawa serotype, we inferred that these non-synonymous mutations did not affect the function of the RfbT transferase.

### Sequence variations in Inaba serotype strains

We sequenced *rfbT* of 74 Inaba isolates from 19 provinces during the 1961–2008 epidemics in China, together with 18 Inaba strains isoloated outside of China (Additional file 1: Table S1). Totally there are 14 classical Inaba strains. Additionally, the sequence of *rfbT* in classical Inaba strain NIH35A3 (accession number X59779) and five other whole genome-sequenced El Tor Inaba strains including N16961 [33], IEC224 [37], MJ-1236 [34], CIRS101 [34] and LMA3984-4 [38]) were obtained from GenBank genome database and added to the comparison. The *rfbT* gene (VCD_001363) of MJ-1236 was recognized as a shorter fragment of 819 bp in its annotation file, we revised the sequence by including a 49 nucleotide region exactly located in the upstream of the originally recognized start codon “TTG” (positions 375973–376021 in the genomic sequence of CP001485.1) in our analysis after sequence examination and alignment.

**Figure 2:**
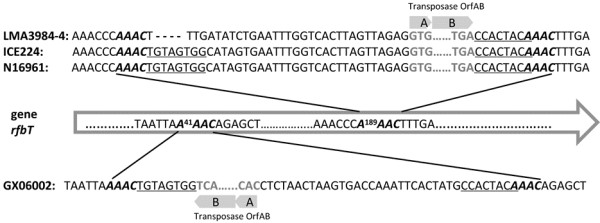
**The sequence alignment of the transposase OrfAB insertion regions in the *rfbT *gene.** The 8-bp terminal inverted repeats are underlined; possible duplicated AAAC insertion sequences are italicized and in bold; the ORFs of the transposase OrfAB element are shown by hollow arrows with the indicated start and end codons.

The sequence comparison of *rfbT* from totally 98 Inaba strains revealed multiplex mutational events (Table 1), which had occurred in 21 positions along the *rfbT* gene. One type of mutation was transposable element mediated. Specifically, an *IS*Vch5 transposase was inserted at the C^49^TTG site of the *rfbT* sequence in strain SD95001, with the 4-bp insertion sequence duplication. A transposase OrfAB gene element was inserted in the *rfbT* genes of strains N16961, IEC224, LMA3984-4 and GX06002. The transposase OrfAB gene contains two partially overlapping open reading frames, with 8 bp terminal inverted repeats (TGTAGTGG/CCACTACA) (Figure 2). It was uniquely inserted at the A^189^AAC site of the *rfbT* coding sequence in N16961, IEC224 and LMA3984-4. In contrast in the GX06002 strain, it was reversely inserted at the A^41^AAC site. Both insertion events duplicated the target sequence which flanked at both sides of transposase OrfAB (Figure 2).

**Table 1:** **Nucleotide sequence changes in the *rfbT *gene of different Inaba strains of *V. cholerae *O1 El Tor relative to El Tor Ogawa strain B33**

**Mutational event**	**Change(s)**	**Strain(s)**	**Consequence**
Transposase insertion	Transposase inserted at 5′ region of *rfbT*	N16961, IEC224, LMA3984-4, GX06002*, SD95001*	RfbT inactivation
Single nucleotide mutation	G-3 to A	ZJ011319*, GD01049, CQ02057, GX01012, JX01002, LN01-1, CQ01008	114 amino acids of N-terminal untranslated
	A-20 deleted	ZJ05070	Frameshift: K^6^NYVQKL to K^6^TMYKN-Stop
	A inserted after G-55	XJ7517, XJ81759*	Frameshift: D^18^AIQSKSVHD to D^18^ DYSVKVCS-Stop
	G −137 to T	569B^§^, 16510^§^, 16186^§^, 16177^§^, 16159^§^, 16156^§^, 16148^§^, 1119^§^, 16020^§^, 16002^§^, 16121^§^, 16505^§^, 16507^§^, NIH35A3^§^	RfbT W^46^ to L
	T-303 deleted	569B^§^, 1119^§^, 16020^§^, 16002^§^, 16505^§^, 16507^§^	RfbT T^102^ to H
	C inserted after C-307	569B^§^, 1119^§^, 16020^§^, 16002^§^, 16505^§^, 16507^§^	
		16121*^§^, 16148^§^, 16510^§^, 16186^§^, 16177^§^, 16159^§^, 16156^§^, NIH35A3^§^	Frameshift: T^102^QGKIIAIEPLTEMENS to T^102^PRKNYSDRTTHRNGK-Stop
	C-307 to T	E506	Frameshift: T^102^Q to T^102^-Stop
	T-472 to C	FJ05234*, ZJ05023, GD05039, HL08091, FJ147, CIRS101	RfbT S^158^ to P
	A-482 to T	XJ05021*	RfbT Y^161^ to F
	C-487 to A	569B^§^, 16510^§^, 16186^§^, 16177^§^, 16159^§^, 16156^§^, 16148^§^, 1119^§^, 16020^§^, 16002^§^, 16121^§^, 16505^§^, 16507^§^, NIH35A3^§^	RfbT Q^163^ to K
	A-494 deleted	16020*^§^, 16002^§^, 16505^§^, 16507^§^, 16121^§^, 1119^§^; GX00107*, XJ05021*, GD91070, GD06119, GD06009, JX04043, GX06021	Frameshift: K^164^NTDI to K^164^IQT-Stop (16121 yields truncated proteins due to nonsense mutations at 307nt)
	A-495 deleted	V01, C6706, X190	Frameshift: K^164^NTDI to K^164^IQT-Stop
	C-563 to A	TJ64600, JS32*, BJ6588,	Frameshift: I^187^S to I^187^-Stop
	G-655 to T	569B^§^	Frameshift: N^218^G to N^218^-Stop
	C-785 to A	LN04060	Frameshift: L^261^S to L^261^-Stop
Multiple nucleotides mutation	TGATGCT inserted after T-57	MJ1236	Frameshift: A^19^I to A^19^-Stop
	TATG insterted after G-207	T21	Frameshift: M^69^RHWIVNH to M^69^YASLDC-Stop
	GACACAT inserted after T-237	HE65441*, BJ84203, JX8672, JS63257	Frameshift: T^81^T to T^81^-Stop
	GCTGAACATCC deleted after C-789	BJ821*, JX801361, BJ83801, FJ85063, HN8232, JS80269, JX801360, SD83101, AH88602, FJ86104, HN81331, JX801309, JX801363, BJ83795, JS80215, JX801305, JX84172, JX8788, SD83163, SD83164, SD83167, SX8429, JX801342, HN84345, FJ80004, GD791080, GD791084, GD861812, HN81175, JX801295, JX8659, JX87123, SC83535, ZJ861071, FJ8004, JX84190, LN85092, SD83176, JX801290, JS80252, FJ85010, ZJ82428	Frameshift: S^263^A to S^263^-Stop

* the strain selected as the representative of the different kinds of mutations and examined the seroconversion in complement assay by inducing a *rfbT*- expressing plasmid, pBR-*rfbT*.

^§^ classical biotype.

Other sequences variations were due to nucleotide mutations, including deletions, insertions and base substitutions (Table 1). Compared to the El Tor biotype Ogawa strain B33, 39.8% (39/98) of the analyzed Inaba strains exhibited single nucleotide mutations (deletion, insertion or substitution) at different positions of the *rfbT* gene which caused a frameshift and resulted in truncated RfbT proteins. Additionally, three particular mutations at positions 137, 472 and 487 were found to cause non-synonymous mutations. The substitutions of G137T and C487A in all 14 classical Inaba strains, compared to the *rfbT* sequence of El Tor Ogawa strain B33, resulted in a replacement of W46L and Q163K, respectively, the same as had been found in the classical Ogawa strains (Additional file 2: Figure S1). The substitution of T472C of *rfbT* in strains FJ05234, ZJ05023, FJ147, GD05039, HL08091 and CIRS101 resulted in a change of S158P of RfbT, which is the only single mutation found in these strains and suggests that a Ser residue at this position is critical for the function of the RfbT transferase. Another important site is position 482, for the resulting Y^161^ to F substitution determined Inaba phenotype in XJ05021 too.

The insertion of C at the position after C-307 occurred in all 14 classical Inaba strains, but caused two different mutations (Table 1, Additional file 2: Figure S1). In strains 16121, 16148, 16510, 16186, 16177, 16159, 16156, and NIH35A3, this insertion caused a frameshift mutation, subsequently formed an internal stop codon after position 348, leading to premature termination of RfbT translation. Whereas in strains 569B, 1119, 16020, 16002, 16505 and 16507, the reading frame was maintained since this insertion was counteracted by the prior deletion at position 303. The combined effect of the two sites of mutations led to the replacement of T102H in these strains. The Inaba phenotype of theses strains resulted from the truncated RfbT caused by an A-494 deletion or G655T substitution (only for 569B) on the posterior sequence. Strains V01, C6706, X190 which were isolated from South America had identical single nucleotide mutation different from E506 isolated from America. In addition, 48.0% (47/98) of the strains were noted to have short-sequence indels (insertions/deletions) resulting in truncated and prematurely-terminated RfbT proteins.

### Characteristic mutations of Inaba strains in the Inaba dominant epidemics in China

**Figure 3:**
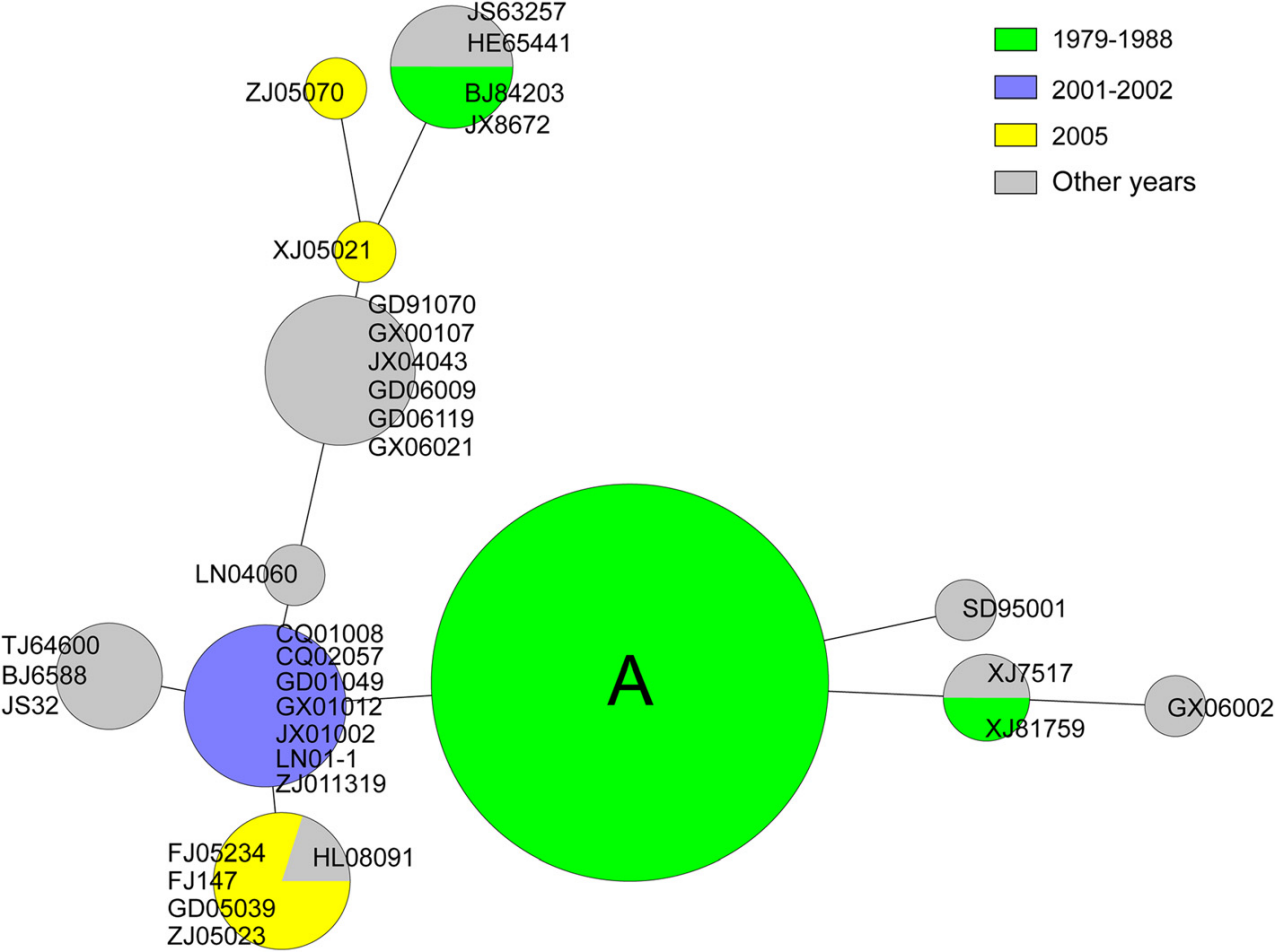
**Minimal spanning tree of 74 Inaba strains of O1 El Tor *V. cholerae *isolated in China based on the *rfbT *sequence.** Subtypes are indicated by circles, whose diameter increases as the number of strains increases. Colors were used to indicate the different time periods when the strains were isolated. The strain names in different subtypes are shown in the corresponding circles except the one labeled A. Strains in circle A were characterized with 11-bp deletion and isolated during the first Inaba dominated epidemic period (1979–1988). Circle A includes strain BJ821, JX801361, BJ83801, FJ85063, HN8232, JS80269, JX801360, SD83101, AH88602, FJ86104, HN81331, JX801309, JX801363, BJ83795, JS80215, JX801305, JX84172, JX8788, SD83163, SD83164, SD83167, SX8429, JX801342, HN84345, FJ80004, GD791080, GD791084, GD861812, HN81175, JX801295, JX8659, JX87123, SC83535, ZJ861071, FJ8004, JX84190, LN85092, SD83176, JX801290, JS80252, FJ85010 and ZJ82428.

During the cholera epidemics from 1961 to 2008 in China, Ogawa/Inaba serotype shifts were observed. Three Inaba serotype dominant multiyear epidemics occurred in 1976–1989, 2001–2002 and around 2005. In this study, most (42/45) Inaba strains isolated in 1979–1988 displayed the identical mutation of 11-bp (GCTGAACATCC) deletion (Figure 3). These strains were isolated from 11 different provinces, and were characterized with the marker mutation of *rfbT* in the epidemics (Table 1). PFGE subtyping on 36 of these 41 strains (Additional file 3: Figure S2) showed that 17 strains possessed the same pattern, while other patterns only displayed 1–3 band differences compared to the predominant pattern, with similarity coefficient values of 93.0%-97.7%, indicating they were closely related in terms of their genetic background. Among three other strains, two showed GACACAT insertion after T-237, one strain had an A insertion after G-55, both caused the frameshift and truncated RfbT proteins (Table 1). These *rfbT* mutations resulted in sporadic Inaba strains in these epidemics.

During 2001–2002 Inaba serotype dominant epidemics, all test seven Inaba strains isolated from six provinces had the same G3A substitution only, which caused disappearance of the start codon (ATG) and thus no translation of the 114 amino acid residues of RfbT N-terminal (Table 1, Figure 3). Among the six Inaba strains isolated in the epidemic of 2005, four showed predominant A472T mutation which resulted in S158P of RfbT, whereas the other two strains (ZJ05070 and XJ05021) had different mutations (Table 1, Figure 3). In different Inaba serotype dominant epidemics the strains had the individually predominant mutations within RfbT. Different *rfbT* mutations were observed among the Inaba strains in the non-Inaba-dominant years (Table 1, Figure 3). The same *rfbT* mutations were sometimes found in the Inaba strains isolated in the non-Inaba-dominant years, even from different countries, such as transposase insertion, A-494 deletion and GACACAT insertion (Table 1), suggesting the hot spots of mutation, or wide distribution of such strains.

### Seroconversion of the Inaba strain containing a *rfbT* expressing-plasmid and construction of T472 C substitution mutant in Ogawa background

To determine the *rfbT* mutations observed in this study were responsible for the serotyping, we induced the seroconversion of Inaba strains by introducing the recombinant plasmid pBR-*rfbT* carrying intact *rfbT* gene. Twelve Inaba strains which contained different frameshifts were selected. Agglutination of the transformed strains with specific typing sera showed that all but one (GX06002) had been converted from Inaba to Ogawa. Interestingly, for strain GX06002, some transformed colonies were converted to Ogawa, while other colonies maintained the Inaba serotype. One possible explanation for this result may be the different copy numbers of the plasmid in the host cells. Chiang *et al.* also reported that the presence of one copy of *rfbT* on the chromosome is not sufficient to restore the Ogawa serotype [31].

To further confirm that single point mutation only causing non-synonymous mutation was truly involved in the serotype shift, we performed the opposite experiment, i. e. induction of Inaba serotype in Ogawa strains. We created the T472C substitution on the chromosomal *rfbT* gene of Ogawa strain 7743 through homologous recombination. As expected, this substitution caused serotype shift from Ogawa to Inaba in strain 7743. Subsequent introducing the recombinant plasmid pBR-*rfbT* carrying intact the *rfbT* gene induced the seroconversion from Inaba to Ogawa phenotype. Taken together, our study experimentally demonstrated T472C substitution is truly involved in the serotype shift.

## Discussion

In this study, we presented the descriptive data regarding cholera serotype-cycling in China over a 48-year (1961–2008) period, and also noted the multiplicity of *rfbT* sequence variations in *V. cholerae* O1 isolates. Three single nucleotide substitutions and deletion mutations of *rfbT* have been reported which caused serotype switching due to a frameshift or crucial amino acid residue change in RfbT [22,41,42]. In our study much more mutations are found in the Inaba serotype strains, including single amino acid residue substitutions, frameshifts caused by single nucleotide and short fragment insertions/deletions, and transposition events. These mutations occurred randomly over the entire open reading frame of *rfbT*, which may suggest the mutations occurred frequently and differently under pressures from environment and human immunity, as well as spontaneous mutation. With the complementation of the intact *rfbT* gene, these Inaba strains were converted to the Ogawa serotype, which validate the mutations on the Inaba serotype conversion.

Our study provides the first evidence that mobile genetic elements, including the transposase OrfAB and *IS*Vch5 transposase, are involved in inactivating the *rfbT* of *V. cholerae*, thus contributing to the serotype interconversion. The insertion of the two kinds of transposases both led to duplication of the inserted sequence. Although there is difference in terms of the insertion position and orientation, the target sequence (AAAC) of the transposase OrfAB elements in different strains was the same. We further surveyed the distribution of transposase OrfAB copies in several strains which genome sequences are available. The copy number and the distribution of transposase OrfAB on chromosomes I and/or II vary in strains from different regions and years. Strain N16961 contains six copies, each chromosome harbors three copies. In IEC224, in addition to the three copies on each chromosome, there is an additional transposase OrfAB subunit B on chromosome I. In strain MJ-1236, all four copies are located on chromosome II. All these and our data suggest that the transposase OrfAB is quite active in transposition in *V. cholerae*, and probably has preferential insertion site(s). For the first time, *IS*5 transposase was found to be involved in the serotype conversion. Two copies of *IS*5 transposase are present on chromosome II of the N16961, 2010EL-1786, M66-2 and IEC224, while in strain SD95001, the *IS*5 transposase inserts into the N-terminal of the *rfbT* gene that was generally located on chromosome I.

The characteristic nucleotide polymorphisms are also observed in the *rfbT* of the classical biotype and El Tor biotype, irrespective of the serotypes. These include G137T, C insertion after C-307 and C487A in all classical strains when compared to El Tor strains (reference sequence is from El Tor Ogawa strain B33, Additional file 2: Figure S1), which suggests that these sites in *rfbT* could be used as nucleotide markers to differentiate both biotypes, as has been shown for other gene alleles, such as *tcpA*, *rstR* and *ctxB*[43-45].

In endemic areas of cholera, it has long been noticed that the dominant serotypes tend to fluctuate, with shifts occurring in the intervals between epidemics of the disease [20,25]. A similar serotype conversion order (Ogawa-Inaba-Ogawa) observed in Bangladesh was found in China. The Ogawa serotype dominated in the early period of the 1960s in China, consistent with a report that the Ogawa serotype was the predominant serotype for a period before 1966 in Bangladesh [20]. The transition of Ogawa to Inaba occurred in 1978 in China, 12 years later than the switch in Bangladesh. After 11 years when the Inaba serotype dominated (1978–1989), the Ogawa serotype again took over the dominance in the 1990s. A similar trend in the prevalence of the Ogawa serotype was also observed in India and Pakistan during almost the same period [41,46].

Questions may raise about the mutations on *rfbT* among the strains in the Inaba dominant epidemics. An 11-bp deletion event was found to be a distinguishable characteristic of Inaba strains during the Inaba dominant ten year period from 1979 to 1988, indicating that these strains may have originated from a common ancestral clone with this mutation, and then disseminated widely during the second epidemic period in China. It was supported by the PFGE fingerprints by showing same or highly similar patterns of these strains, which may have been caused by the minor variations accumulated gradually in such a clone during its long epidemic history (Additional file 3: Figure S2). Such deletion may be mediated by homologous recombination of a 5-bp repeat sequence (*CATCC***GCTGAA*CATCC*** changed to *CATCC*, where the nucleotides in bold indicate the sequence deleted, and the italicized nucleotides are the repeated sequence).

Predominant mutations of *rfbT* were also observed in the Inaba strains during Inaba dominant epidemic years of 2001–2002 and 2005. Inaba strains in 2001–2002 had the single nucleotide substitution which changed the start codon ATG to ATA, complementation of intact *rfbT* into such strain converted the serotype to be Ogawa, showing the role of this nucleotide mutation. T472C was the only nonsynonymous mutation that accounts for a serotype shift in the Inaba strains in 2005, and we experimentally demonstrated the critical role of a serine at this site for the function of RfbT. The same single substitution was also reported in the Inaba strains isolated during different years (2005–2008) in Iran [42] and India [41].

Characteristic *rfbT* mutations occurred in different Inaba serotype dominant epidemics, which may suggest the clonality of the epidemic strains. These mutations can be used as the sequence signatures in the clonal and evolutionary analysis, and even the tracking markers in epidemiological investigations. Serotype conversion and serotype-shifting in cholera epidemics have been thought to be related to the immune response of individuals and the immune status of the overall population, and has also been documented in animal models [20,22,26]. Thereby it could be deduced that in the cholera endemic regions *rfbT* mutation will be an advantage for the spread of Inaba strains following Ogawa serotype epidemic. In general, the conversion of serotype from Ogawa to Inaba is easy to occur, which is simply a *rfbT* mutant enrichment procedure [22]. While the reciprocal serotype conversion, from Inaba to Ogawa, is much more difficult considering the requirement of the reversion of the original mutation and the great variety of the *rfbT* genetic status of Inaba strains. Maybe, the Inaba strains caused by transposase insertion could be relatively liable to reverse to Ogawa phenotype due to the active mobile ability of the insertion element. Some strains were noticed to have accumulated multiple mutations, it remains a puzzle if this represents a transitional state of overcoming the original mutation by introducing the second or third mutation.

## Conclusion

Our study presents the *rfbT* sequence variations of *V. cholerae* O1 isolates during the serotype shifts over a 48-year period in China. Different types of mutational events and new mutation sites resulting in abnormal translation of *rfbT* are observed, and characteristic *rfbT* mutations in different Inaba serotype dominant epidemic periods are found. These distinguishable mutations can be used as the tracing markers in the epidemic clone analysis, and even surveillance for dissemination of specific clones. The *rfbT* mutation and subsequent serotype shifts of the epidemic strains also could be considered as one type of adaption to population immunity barrier in the cholera endemic regions.

## Abbreviations

CT: Cholera toxin; LB: Luria-Bertani; PFGE: Pulsed-field gel electrophoresis; UPGMA: Arithmetic mean algorithm.

## Competing interests

The authors declare that they have no competing interests.

## Authors’ contributions

LW and XZ carried out the molecular genetic studies and participated in the sequence alignment. PL performed gene complementary test. HZ and JZ participated in the PFGE analysis and sequence submission. BK conceived of the study and helped to draft the manuscript. LZ contributed in the strains’ identification and storage. WL participated in the study design and coordination and drafted the manuscript. All authors read and approved the final manuscript.

## Supplementary Material

Additional file 1: Table S1 Information of O1 *V. cholerae* strains used in this study. Click here for file

Additional file 2: Figure S1 The *rfbT* sequence alignment of the mutation sites between the classical and El Tor biotypes. *rfbT* sequences of all classical biotype strains were included in the analysis. For the El Tor biotype strain, a representative sequence of the Ogawa serotype and each mutation in the Inaba serotype are shown. The dots indicate sequence identity. The nucleotides positions are shown. CVC and EVC represent the classical and El Tor biotype *V. cholerae* strains, respectively. * indicates the reconstructed *rfbT* in N16961 was used by removing the insertion sequence of transposase orfAB. Click here for file

Additional file 3: Figure S2 The results of the PFGE analysis using *Not*I digestion of strains characterized by an 11-bp deletion mutation in *rfbT*. The dendrogram was produced using the Dice coefficient and the unweighted-pair group method with an arithmetic mean algorithm (UPGMA) with a position tolerance of 1.3%. Click here for file
