# Supplementary material for: Sequence polymorphisms of rfbT among the Vibrio cholerae O1 strains in the Ogawa and Inaba serotype shifts
Source: BMC Microbiol. 2013 Jul 26;13:173. doi: 10.1186/1471-2180-13-173 (PMC3727987; doi:10.1186/1471-2180-13-173)
Supplement: Additional file 1: Table S1 — Information of O1 V. cholerae strains used in this study. [file 1471-2180-13-173-S1.doc]

**Supplemental table 1. Information of *V. cholerae* strains used in this study.**

| **Strain** | **Biotype** | **Serotype** | **Country/Province** | **Year** | **Source** |
| --- | --- | --- | --- | --- | --- |
| 16503 | classical | Ogawa | Bangladesh | 1964 | Patient |
| 16502 | classical | Ogawa | Bangladesh | 1964 | Patient |
| 16017 | classical | Ogawa | Bangladesh | 1964 | Patient |
| 16113 | classical | Ogawa | Bangladesh | Unknown | Unknown |
| O395 | classical | Ogawa | India | 1965 | Patient |
| Z66 | classical | Ogawa | Zhejiang, China | 1963 | Unknown |
| 63-12 | El Tor | Ogawa | Indonesia | 1961 | Patient |
| 863 | El Tor | Ogawa | Mauritania | 1986 | Patient |
| 6310 | El Tor | Ogawa | Indonesia | 1961 | Patient |
| 6311 | El Tor | Ogawa | Indonesia | 1961 | Patient |
| C7258 | El Tor | Ogawa | Peru | 1991 | Patient |
| 19-22 | El Tor | Ogawa | ZheJiang, China | 1977 | Patient |
| 7743 | El Tor | Ogawa | Shandong, China | 1977 | Environment |
| BJ8212 | El Tor | Ogawa | Beijing, China | 1982 | Environment |
| CQ92081 | El Tor | Ogawa | Chongqing, China | 1992 | Unknown |
| CQ98287 | El Tor | Ogawa | Chongqing, China | 1998 | Environment |
| CQ99049 | El Tor | Ogawa | Chongqing, China | 1999 | Patient |
| GD050111 | El Tor | Ogawa | Guangdong, China | 2005 | Patient |
| GD06133 | El Tor | Ogawa | Guangdong, China | 2006 | Environment |
| GD612533 | El Tor | Ogawa | Guangdong, China | 1961 | Patient |
| GD612633 | El Tor | Ogawa | Guangdong, China | 1961 | Patient |
| GD612639 | El Tor | Ogawa | Guangdong, China | 1961 | Unknown |
| GD612749 | El Tor | Ogawa | Guangdong, China | 1961 | Patient |
| GD61D118 | El Tor | Ogawa | Guangdong, China | 1961 | Patient |
| GD642341 | El Tor | Ogawa | Guangdong, China | 1964 | Patient |
| GD90184 | El Tor | Ogawa | Guangdong, China | 1990 | Unknown |
| GX00020 | El Tor | Ogawa | Guangxi, China | 2000 | Patient |
| HE7925 | El Tor | Ogawa | Hebei, China | 1979 | Unknown |
| HE84225 | El Tor | Ogawa | Hebei, China | 1984 | Patient |
| HA79341 | El Tor | Ogawa | Henan, China | 1979 | Environment |
| JS05002 | El Tor | Ogawa | Jiangsu, China | 2005 | Environment |
| JS73767 | El Tor | Ogawa | Jiangsu, China | 1973 | Unknown |
| JS74538 | El Tor | Ogawa | Jiangsu, China | 1974 | Patient |
| LN04046 | El Tor | Ogawa | Liaoning, China | 2004 | Patient |
| LN04455 | El Tor | Ogawa | Liaoning, China | 2004 | Environment |
| LN8577 | El Tor | Ogawa | Liaoning, China | 1985 | Patient |
| LN93060 | El Tor | Ogawa | Liaoning, China | 1993 | Environment |
| LN93097 | El Tor | Ogawa | Liaoning, China | 1993 | Patient |
| LN93100 | El Tor | Ogawa | Liaoning, China | 1993 | Patient |
| LN98003 | El Tor | Ogawa | Liaoning, China | 1998 | Patient |
| SC00181 | El Tor | Ogawa | Sichuan, China | 2000 | Environment |
| SD75177 | El Tor | Ogawa | Shandong, China | 1975 | Environment |
| SD75178 | El Tor | Ogawa | Shandong, China | 1975 | Environment |
| SD77264 | El Tor | Ogawa | Shandong, China | 1977 | Environment |
| SD7786 | El Tor | Ogawa | Shandong, China | 1977 | Environment |
| SD78445 | El Tor | Ogawa | Shandong, China | 1978 | Environment |
| SD83127 | El Tor | Ogawa | Shandong, China | 1983 | Unknown |
| SH65930 | El Tor | Ogawa | Shanghai, China | 1965 | Screw |
| SX82741 | El Tor | Ogawa | Shanxi, China | 1982 | Environment |
| TJ64193 | El Tor | Ogawa | Tianjing, China | 1964 | Patient |
| TJ84163 | El Tor | Ogawa | Tianjing, China | 1984 | Patient |
| TJ8533 | El Tor | Ogawa | Tianjing, China | 1985 | Patient |
| V05-18 | El Tor | Ogawa | Guangdong, China | 2005 | Animal |
| V0550 | El Tor | Ogawa | Guangdong, China | 2005 | Patient |
| XJ04064 | El Tor | Ogawa | Xinjiang, China | 2004 | Environment |
| XJ04065 | El Tor | Ogawa | Xinjiang, China | 2004 | Unknown |
| XJ642381 | El Tor | Ogawa | Xinjiang, China | 1964 | Patient |
| XJ6637 | El Tor | Ogawa | Xinjiang, China | 1966 | Unknown |
| XJ6674 | El Tor | Ogawa | Xinjiang, China | 1966 | Unknown |
| XJ730398 | El Tor | Ogawa | Xinjiang, China | 1973 | Unknown |
| XJ73107 | El Tor | Ogawa | Xinjiang, China | 1973 | Unknown |
| XJ81739 | El Tor | Ogawa | Xinjiang, China | 1981 | Patient |
| XJ81757 | El Tor | Ogawa | Xinjiang, China | 1981 | Environment |
| XJ98171 | El Tor | Ogawa | Xinjiang, China | 1998 | Patient |
| ZJ62031 | El Tor | Ogawa | Zhejiang, China | 1962 | Patient |
| ZJ62041 | El Tor | Ogawa | Zhejiang, China | 1962 | Patient |
| ZJ62110 | El Tor | Ogawa | Zhejiang, China | 1962 | Patient |
| ZJ75460 | El Tor | Ogawa | Zhejiang, China | 1975 | Unknown |
| ZJ75525 | El Tor | Ogawa | Zhejiang, China | 1975 | Unknown |
| ZJ76423 | El Tor | Ogawa | Zhejiang, China | 1976 | Unknown |
| ZJ781055 | El Tor | Ogawa | Zhejiang, China | 1978 | Unknown |
| 16505 | classical | Inaba | Bangladesh | 1964 | Patient |
| 16507 | classical | Inaba | Bangladesh | 1964 | Patient |
| 1119 | classical | Inaba | India | 1964 | Patient |
| 16002 | classical | Inaba | Bangladesh | Unknown | Unknown |
| 16020 | classical | Inaba | Bangladesh | Unknown | Unknown |
| 16121 | classical | Inaba | Bangladesh | Unknown | Unknown |
| 16148 | classical | Inaba | Bangladesh | Unknown | Unknown |
| 16156 | classical | Inaba | Bangladesh | Unknown | Unknown |
| 16159 | classical | Inaba | Bangladesh | Unknown | Unknown |
| 16177 | classical | Inaba | Bangladesh | Unknown | Unknown |
| 16186 | classical | Inaba | Bangladesh | Unknown | Unknown |
| 16510 | classical | Inaba | Bangladesh | 1980 | Patient |
| 569B | classical | Inaba | India | 1948 | Patient |
| C6706 | El Tor | Inaba | Peru | 1991 | Patient |
| E506 | El Tor | Inaba | America | Unknown | Unknown |
| T21 | El Tor | Inaba | Thailand | 1990 | Unknown |
| V01 | El Tor | Inaba | Chile | 1991 | Patient |
| X190 | El Tor | Inaba | Peru | 1991 | Patient |
| BJ83801 | El Tor | Inaba | Beijing, China | 1983 | Unknown |
| BJ84203 | El Tor | Inaba | Beijing, China | 1984 | Unknown |
| CQ01008 | El Tor | Inaba | Chongqing, China | 2001 | Patient |
| CQ02057 | El Tor | Inaba | Chongqing, China | 2002 | Patient |
| FJ05234 | El Tor | Inaba | Fujian, China | 2005 | Patient |
| FJ147 | El Tor | Inaba | Fujian, China | 2005 | Patient |
| FJ80004 | El Tor | Inaba | Fujian, China | 1980 | Patient |
| FJ8004 | El Tor | Inaba | Fujian, China | 1980 | Environment |
| FJ85010 | El Tor | Inaba | Fujian, China | 1985 | Patient |
| FJ85063 | El Tor | Inaba | Fujian, China | 1985 | Patient |
| FJ86104 | El Tor | Inaba | Fujian, China | 1986 | Patient |
| GD01049 | El Tor | Inaba | Guangdong, China | 2001 | Patient |
| GD05039 | El Tor | Inaba | Guangdong, China | 2005 | Patient |
| GD06009 | El Tor | Inaba | Guangdong, China | 2006 | Environment |
| GD06119 | El Tor | Inaba | Guangdong, China | 2006 | Environment |
| GD791080 | El Tor | Inaba | Guangdong, China | 1979 | Patient |
| GD791084 | El Tor | Inaba | Guangdong, China | 1979 | Patient |
| GD861812 | El Tor | Inaba | Guangdong, China | 1986 | Patient |
| GD91070 | El Tor | Inaba | Guangdong, China | 1991 | Patient |
| GX00107 | El Tor | Inaba | Guangxi, China | 2000 | Patient |
| GX01012 | El Tor | Inaba | Guangxi, China | 2001 | Patient |
| GX06002 | El Tor | Inaba | Guangxi, China | 2006 | Environment |
| GX06021 | El Tor | Inaba | Guangxi, China | 2006 | Environment |
| HE65441 | El Tor | Inaba | Hebei, China | 1965 | Environment |
| HL08091 | El Tor | Inaba | Heilongjiang, China | 2008 | Patient |
| HN81175 | El Tor | Inaba | Hunan, China | 1981 | Patient |
| HN81331 | El Tor | Inaba | Hunan, China | 1981 | Fly |
| HA8232 | El Tor | Inaba | Henan, China | 1982 | Patient |
| HA84345 | El Tor | Inaba | Henan, China | 1984 | Patient |
| JS32 | El Tor | Inaba | Jiangsu, China | 1990 | Patient |
| JS63257 | El Tor | Inaba | Jiangsu, China | 1963 | Environment |
| JS80215 | El Tor | Inaba | Jiangsu, China | 1980 | Patient |
| JS80252 | El Tor | Inaba | Jiangsu, China | 1980 | Patient |
| JS80269 | El Tor | Inaba | Jiangsu, China | 1980 | Patient |
| JX01002 | El Tor | Inaba | Jiangxi, China | 2001 | Patient |
| JX04043 | El Tor | Inaba | Jiangxi, China | 2001 | Turtle |
| JX801290 | El Tor | Inaba | Jiangxi, China | 1980 | Patient |
| JX801295 | El Tor | Inaba | Jiangxi, China | 1980 | Patient |
| JX801305 | El Tor | Inaba | Jiangxi, China | 1980 | Environment |
| JX801309 | El Tor | Inaba | Jiangxi, China | 1980 | Patient |
| JX801342 | El Tor | Inaba | Jiangxi, China | 1980 | Unknown |
| JX801360 | El Tor | Inaba | Jiangxi, China | 1980 | Patient |
| JX801361 | El Tor | Inaba | Jiangxi, China | 1980 | Patient |
| JX801363 | El Tor | Inaba | Jiangxi, China | 1980 | Environment |
| JX84172 | El Tor | Inaba | Jiangxi, China | 1984 | Patient |
| JX84190 | El Tor | Inaba | Jiangxi, China | 1984 | Patient |
| JX8659 | El Tor | Inaba | Jiangxi, China | 1986 | Patient |
| JX8672 | El Tor | Inaba | Jiangxi, China | 1986 | Environment |
| JX87123 | El Tor | Inaba | Jiangxi, China | 1987 | Environment |
| JX8788 | El Tor | Inaba | Jiangxi, China | 1987 | Patient |
| LN01-1 | El Tor | Inaba | Liaoning, China | 2001 | Patient |
| LN04060 | El Tor | Inaba | Liaoning, China | 2004 | Patient |
| LN04547 | El Tor | Inaba | Liaoning, China | 2004 | Environment |
| SC83535 | El Tor | Inaba | Sichuan, China | 1983 | Clinical |
| SD83101 | El Tor | Inaba | Shandong, China | 1983 | Unknown |
| SD83163 | El Tor | Inaba | Shandong, China | 1983 | Unknown |
| SD83164 | El Tor | Inaba | Shandong, China | 1983 | Unknown |
| SD83167 | El Tor | Inaba | Shandong, China | 1983 | Unknown |
| SD83176 | El Tor | Inaba | Shandong, China | 1983 | Unknown |
| SD95001 | El Tor | Inaba | Shandong, China | 1995 | Unknown |
| SN8429 | El Tor | Inaba | Shanxi, China | 1984 | Unknown |
| TJ64600 | El Tor | Inaba | Tianjing, China | 1964 | Environment |
| XJ05021 | El Tor | Inaba | Xinjiang, China | 2005 | water |
| XJ7517 | El Tor | Inaba | Xinjiang, China | 1975 | Environment |
| XJ81759 | El Tor | Inaba | Xinjiang, China | 1981 | Environment |
| ZJ011319 | El Tor | Inaba | Zhejiang, China | 2001 | Environment |
| ZJ05023 | El Tor | Inaba | Zhejiang, China | 2005 | Patient |
| ZJ05070 | El Tor | Inaba | Zhejiang, China | 2005 | Environment |
| ZJ82428 | El Tor | Inaba | Zhejiang, China | 1982 | Patient |
| ZJ861071 | El Tor | Inaba | Zhejiang, China | 1986 | Patient |
