# Supplementary figures and images for: Sequence polymorphisms of rfbT among the Vibrio cholerae O1 strains in the Ogawa and Inaba serotype shifts
Source: BMC Microbiol. 2013 Jul 26;13:173. doi: 10.1186/1471-2180-13-173 (PMC3727987; doi:10.1186/1471-2180-13-173)

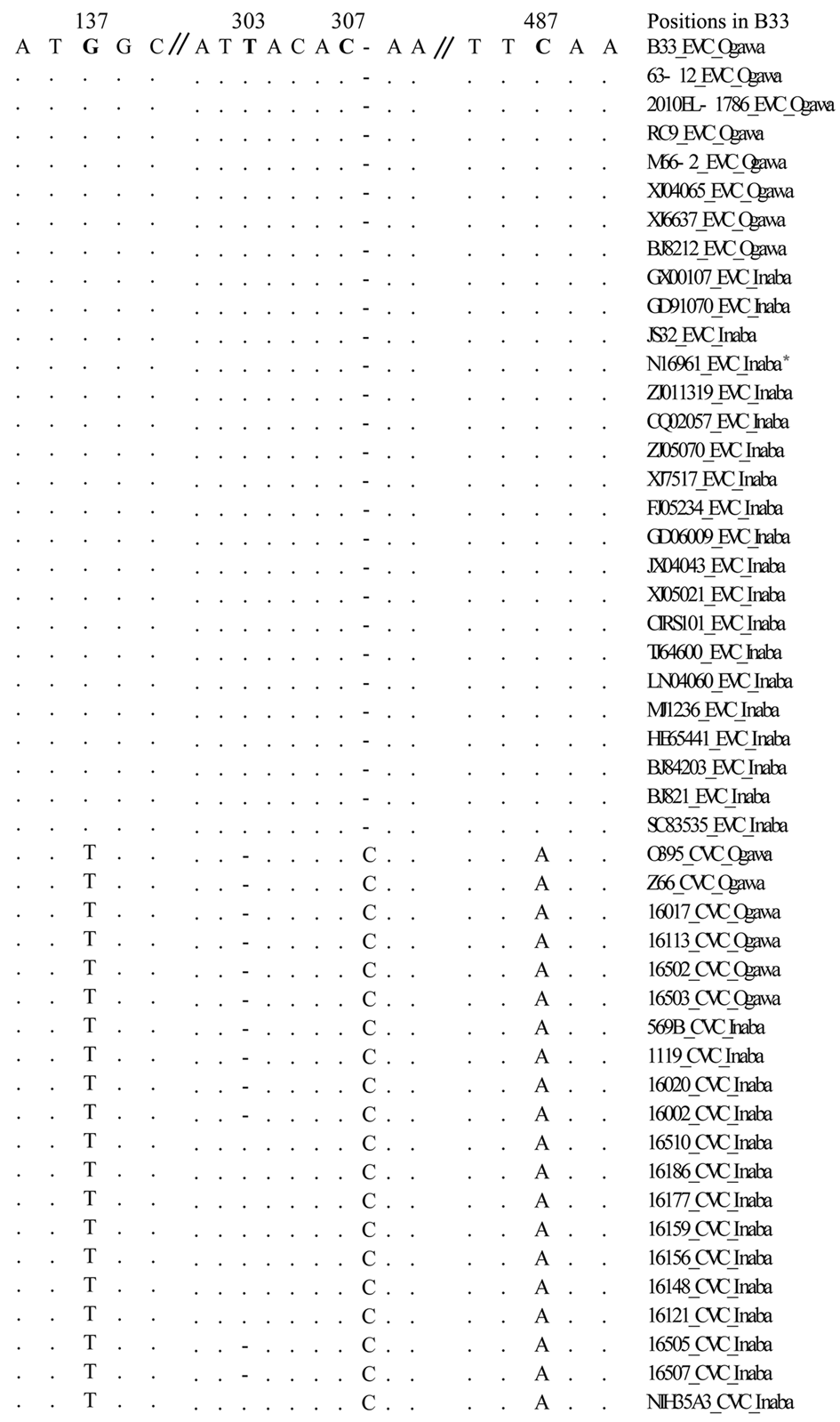

Supplement: Additional file 2: Figure S1 — The rfbT sequence alignment of the mutation sites between the classical and El Tor biotypes. rfbT sequences of all classical biotype strains were included in the analysis. For the El Tor biotype strain, a representative sequence of the Ogawa serotype and each mutation in the Inaba serotype are shown. The dots indicate sequence identity. The nucleotides positions are shown. CVC and EVC represent the classical and El Tor biotype V. cholerae strains, respectively. * indicates the reconstructed rfbT in N16961 was used by removing the insertion sequence of transposase orfAB. [file 1471-2180-13-173-S2.tiff]

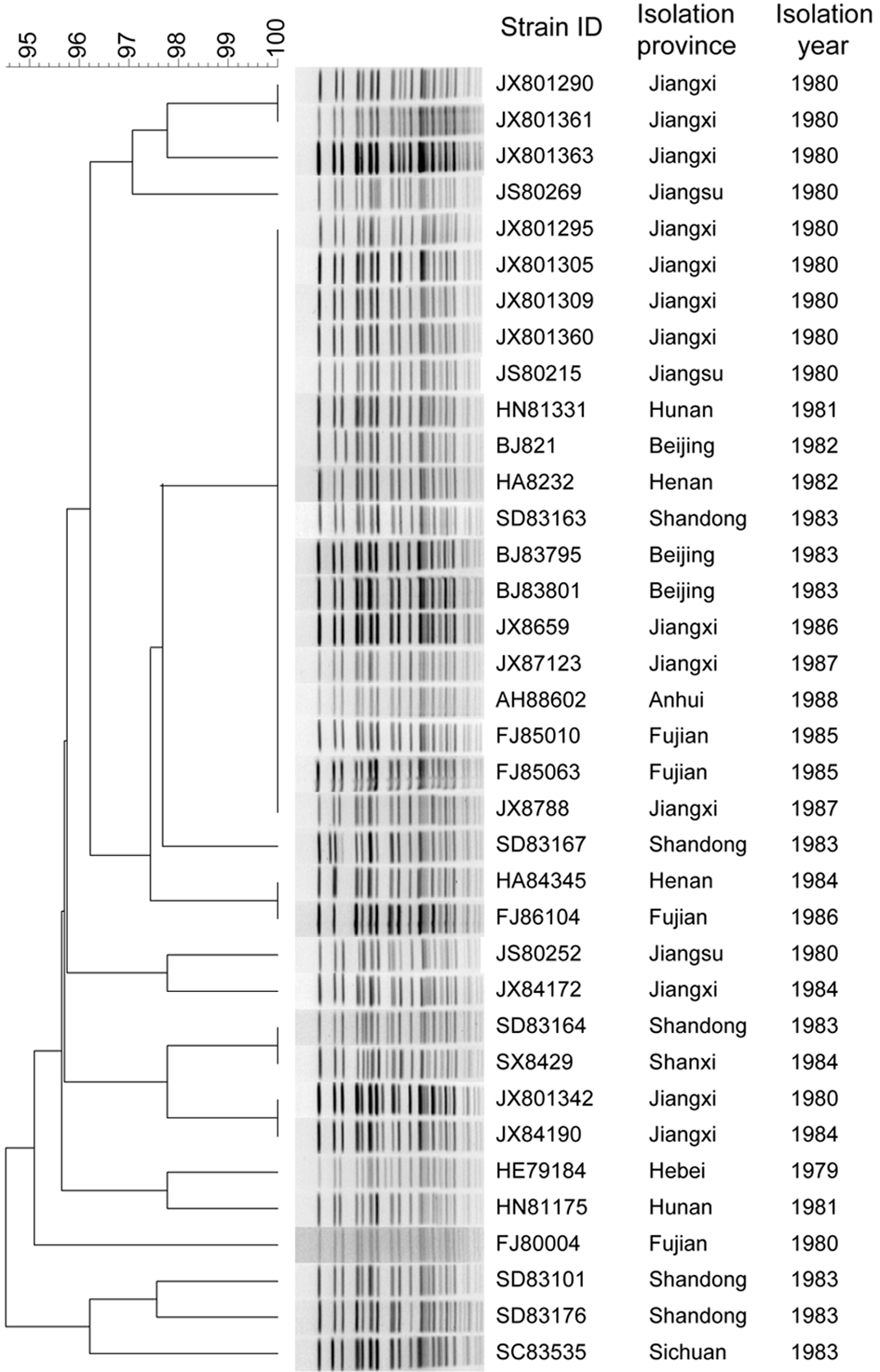

Supplement: Additional file 3: Figure S2 — The results of the PFGE analysis using NotI digestion of strains characterized by an 11-bp deletion mutation in rfbT. The dendrogram was produced using the Dice coefficient and the unweighted-pair group method with an arithmetic mean algorithm (UPGMA) with a position tolerance of 1.3%. [file 1471-2180-13-173-S3.tiff]
